# Modulation of mTORC1 Signaling Pathway by HIV-1

**DOI:** 10.3390/cells9051090

**Published:** 2020-04-28

**Authors:** Burkitkan Akbay, Anna Shmakova, Yegor Vassetzky, Svetlana Dokudovskaya

**Affiliations:** 1 CNRS UMR 9018, Université Paris-Saclay, Institut Gustave Roussy, 114, rue Édouard Vaillant, 94805 Villejuif, France; akbayburkitkan@gmail.com (B.A.); anyashm@gmail.com (A.S.); yegor.vassetzky@cnrs.fr (Y.V.); 2 LIA 1066 LFR2O French-Russian Joint Cancer Research Laboratory, 114, rue Édouard-Vaillant, 94805 Villejuif, France; 3 Koltzov Institute of Developmental Biology, 26, Vavilova str., 119334 Moscow, Russia

**Keywords:** HIV-1, HIV-1 related diseases, mTORC1 pathway, autophagy

## Abstract

Mammalian target of rapamycin complex 1 (mTORC1) is a master regulator of cellular proliferation and survival which controls cellular response to different stresses, including viral infection. HIV-1 interferes with the mTORC1 pathway at every stage of infection. At the same time, the host cells rely on the mTORC1 pathway and autophagy to fight against virus replication and transmission. In this review, we will provide the most up-to-date picture of the role of the mTORC1 pathway in the HIV-1 life cycle, latency and HIV-related diseases. We will also provide an overview of recent trends in the targeting of the mTORC1 pathway as a promising strategy for HIV-1 eradication.

## 1. Introduction

Viruses generate an environment that is favorable for their successful replication and transmission during infection. In order to optimize their biosynthetic needs, viruses use the nutrient, energy and macromolecule synthesis systems of the host cells and manipulate their metabolism [1]. Host cells in turn respond to viral infection by changing their transcriptional and translational programs and employing antiviral metabolic changes [2,3,4]. Cellular response to various stresses, including viral infection, is under the control of the mechanistic target of rapamycin complex 1 (mTORC1), which drives proliferation and survival by the regulation of anabolic and catabolic processes. Thus, it is no wonder that viruses try to use this signaling pathway to their benefit [4].

The human immunodeficiency virus type-1 (HIV-1) is a lentivirus containing two positive-sense single strand RNAs encapsulated in a capsid formed by p24. Structural HIV-1 proteins (Gag, Pol and Env) are produced as polypeptides and subsequently processed into matrix proteins, protease, reverse transcriptase, integrase and surface proteins gp120 and gp41. HIV-1 also codes for two regulatory components: Tat (transcriptional trans-activator) and Rev (regulator of expression of virion proteins). Finally, Vpr, Vif, Nef and Vpu serve as accessory regulatory elements [5]. During viral entry, gp120 binds to the CD4 molecule of the host cell and gp41 binds to the cellular coreceptors such as CCR5 and CXCR4. After fusion with the host cell, a conical capisid around the HIV-1 genome disassembles (a process known as uncoating), and viral RNA is released into the cytoplasm where it is transcribed by a viral-encoded reverse transcriptase. Uncoating probably occurs in the cytoplasm in coordination with reverse transcription or at the nuclear envelope during nuclear import. Subsequently, viral dsDNA uses the host nuclear import machinery to move to the host cell nucleus, where it integrates into the host DNA with the help of a viral-encoded integrase. Remarkably, recent studies revealed that intact viral cores can enter to the nucleus and uncoat just before integration to their chromosomal integration sites [6]. Pro-viruses use the host RNA polymerase to synthetize mRNA, which is subsequently translated into viral proteins. HIV-1 infects and kills cells of the immune system such as T-helper cells, macrophages and dendritic cells, leading to immunodeficiency and further increasing the incidence of opportunistic infections and cancers.

The mechanistic target of rapamycin (mTOR) is an evolutionarily-conserved, serine-threonine protein kinase that belongs to the phosphatidylinositol 3-kinase PI3K-related family. mTOR forms two different macromolecular protein complexes, mTORC1 and mTORC2, which differ in their composition, downstream targets and regulation [7]. mTORC1 is sensitive, while mTORC2 is much less responsive to an allosteric mTOR inhibitor rapamycin (Sirolimus^®^), an immunosuppressor, which suppresses T and B cell activation by inhibition of the cell cycle. Various analogues of rapamycin, so called rapalogues (Everolimus^®^, Temsirolimus^®^), are also frequently used in clinics for immunosuppression. In addition, a number of alternative mTOR inhibitors have been developed. These inhibitors block both mTORC1 and mTORC2 (pan-inhibitors or TOR-KIs, i.e., INK128) or act on mTOR kinase and another protein (dual inhibitors), most often targeting a network upstream of mTORC1/2 [8]. Viruses are the leading cause of infections after solid-organ transplant and during anticancer treatment; the use of mTOR inhibitors decreases the incidence of viral infection in these medical conditions [9,10,11].

One of the first pieces of evidence that mTORC1 was involved in HIV-1 infection came from the observation that treatment with rapamycin causes downregulation of CCR5 expression in T cells [12]. A number of studies that immediately followed confirmed that rapamycin possessed anti-HIV-1 properties both in vitro and in vivo, pointing to the mTORC1 importance during HIV-1 propagation (reviewed in [13,14]). Pan-inhibitors of mTORC1 block HIV-1 even more efficiently, interfering both with virus entry (by reducing CCR5 levels) and with basal and induced transcription, as shown in preclinical humanized mice models [15]. Our review is focused on recently discovered mechanisms of mTORC1 contribution to HIV-1 infection, latency and development of HIV-1 related diseases.

## 2. mTORC1, a Main Metabolic Network of the Cell

mTORC1 integrates signals from many intracellular and extracellular cues: amino acids, growth factors, energy, oxygen, DNA damage and infectious agents, including viruses. Depending on the nature of the signal, its duration, cell type and many other factors, mTORC1 will “determine” the subsequent cell fate. mTORC1 can accelerate proliferation via the phosphorylation of its key targets, p70S6 Kinase 1 (S6K1) and members of eIF4E Binding Protein family (4E-BPs), which participate in the synthesis of three major cell constituents: proteins, nucleotides and lipids [7]. Alternatively, mTORC1 can drive the cell through a catabolic process, since it controls both autophagy (via phosphorylation of ULK1 and TFEB) and protein degradation by ubiquitin-proteasome system (via phosphorylation of ERK5) [16]. In order to operate through signals and responses, mTORC1 manipulates one of the most complex signaling networks in the cell, the mTORC1 pathway.

**Figure 1:**
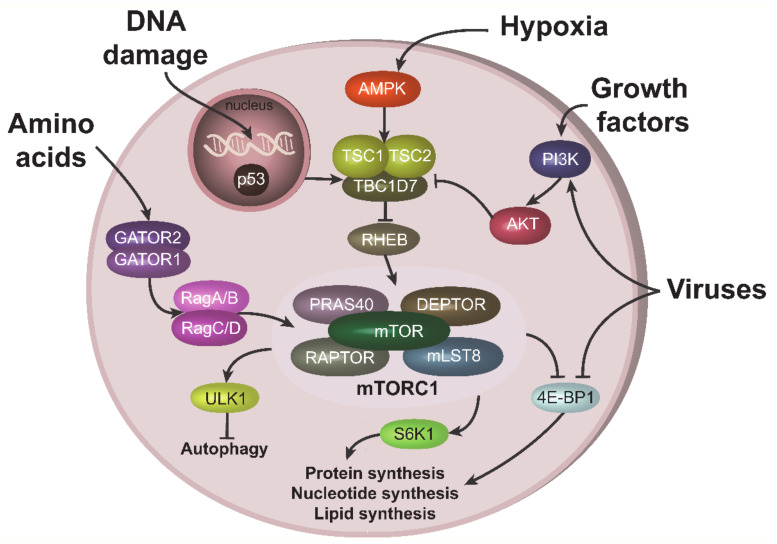
Upstream and downstream mTORC1 signaling. mTORC1 can be stimulated by various cues. Amino acids activate mTORC1 through GATOR1/GATOR2 complexes and RAGs. Growth factors recruits PI3K to the plasma membrane, which results in subsequent AKT activation. AKT can stimulate mTORC1 either directly through the phosphorylation of its component PRAS40, or via phosphorylation and inhibition of TSC1 from the TSC complex, which leads to RHEB and mTORC1 activations. Under hypoxic condition, mTORC1 is repressed due to TSC activation via AMPK. TSC can also be stimulated (i.e., mTORC1 repressed) via p53 pathway during DNA damage. Viruses can activate mTORC1, especially at the late stage of infection, both upstream, via PI3K/AKT, or acting on downstream mTORC1 target 4E-BP1.

Growth factors and cytokines activate mTORC1 through the PI3K-AKT signaling pathway [17]. Signals from these stimuli activate serine-threonine kinase AKT, leading to the phosphorylation and inactivation of the TSC2 component of tuberous sclerosis complex (TSC), an mTORC1 inhibitor. TSC inactivation results in the stimulation of RHEB GTPase, which activates mTORC1 [18] (Figure 1). AKT can also activate mTORC1 directly, in a TSC-independent way, by phosphorylating and inactivating the mTORC1 inhibitory component, PRAS40 [19]. Amino acids activate mTORC1 through a number of specific amino acid sensors. These sensors in turn stimulate GATOR1/GATOR2 complexes, containing a number of proteins, including tumor suppressors such as DEPDC5 [20]. Stimulation of GATORs further induces Rag GTPases (RagA/B and RagC/D), leading to mTORC1 activation (Figure 1) [21]. In contrast, low energy levels and hypoxic conditions result in mTORC1 inhibition via stimulation of AMP-activated protein kinase (AMPK) [22,23]. DNA damaging agents also suppress mTORC1 activity, in part by activation of the most powerful mTORC1 antagonist, p53 (Figure 1) [8,24].

Viruses can both activate and inhibit the mTORC1 pathway, depending on the type of the virus, stage on infection and cells in contact with the virus (reviewed in [4]). HIV-1 infection is generally associated with inhibition of mTORC1 in CD4 T cells at the early stage of infection and the activation of mTORC1 at the late stage (see below). This behavior is often related with the ability of mTORC1 to inhibit autophagy. Indeed, at the early state of infection, host cells try to activate autophagy and/or apoptosis to eliminate the spread of the virus; therefore, they need to suppress mTORC1 activity. In contrast, at the late state of infection, when host cells have “lost the battle”, viruses try to activate mTORC1 in order to suppress autophagy. Interestingly, viruses can target the mTORC1 pathway both upstream and downstream of mTORC1. For instance, Kaposi’s sarcoma-associated herpes virus (KSHV), related to the AIDS defining cancer of the same name, activates mTORC1 upstream through the PI3K-AKT axis [25], while herpesviruses can also act downstream of the mTORC1 node, mainly through stimulation of phosphorylation and inactivation of 4E-BP1 (Figure 1) [4].

## 3. mTORC1 in the Immune System

mTORC1 plays a central role in the metabolism, differentiation and effector functions of immune cells [26,27]. Here, we will only briefly describe the role of mTORC1 in B and T lymphocytes, as they are the most relevant during HIV-1 infection and in the development of HIV-1-related diseases (e.g., B-cell lymphomas). Many more details can be found in exhaustive recent reviews about the role of mTORC1 in B cells [28], T cells [29] and NK, macrophages and dendritic cells [30].

T lymphocytes in the resting state are catabolic and use autophagy to produce molecules necessary for protein synthesis and energy. After activation, T cells become anabolic and switch to glycolysis to produce energy and various substrates for proliferation. A transition from the resting to active state requires the upregulation of metabolic pathways controlled by mTORC1.

CD4 T cells, which are the primary target of HIV-1, are effector T cells which can be differentiated into distinct effector lineages (Th1, Th2, Th17) in response to various inflammatory cytokines. mTORC1 helps this differentiation process by promoting glycolysis and lipid biosynthesis [31,32,33,34]. mTORC1-deficient T cells have an impaired ability to differentiate [31]. Similarly, T cells lacking the mTORC1 activator RHEB also fail to differentiate into Th1 and Th17 [32]. Alternatively, the deletion of mTORC1 inhibitor TSC1 results in enhanced mTORC1 activity, leading to elevated Th1 and Th17 differentiation and multiorgan inflammation in mice [35]. In addition, a deficiency in the leucine transporter LAT1 [36] and glutamine transporter ASCT2 [37] impairs Th1 and Th17 differentiation in a mTORC1-dependent manner.

CD8 T cells have the ability to kill cancer cells or virally-infected cells (effector cytotoxic T cells), and maintain long-term memory (memory T cells). Increased mTORC1 activity is beneficial for the generation of effector CD8 T cells. In contrast, active mTORC1 downregulates memory T cell formation, resulting in a decreased response to secondary immunization [38,39,40].

Tregs are immune cells that negatively control both cytotoxic effector CD8 T cells and effector CD4 T cells. mTORC1 activity has negative effects on Treg generation [41], while it is positively correlated with the inhibitory functions of Tregs towards other T cells [42]. Active mTORC1 promotes the conversion of Tregs to effector-like T cells and further impairs Treg stability and function [35,43,44]. Toll-like receptor signals, which promote Treg proliferation, increase mTORC1 signaling, glycolysis and expression of glucose transporter Glut1 [43].

B cells undergo multiple steps of maturation before becoming plasma cells and acquiring antibody-secreting capacity, which is critical for defense against recurrent infections. mTORC1 is highly active at the earliest stages of B cell development, and its activity reduces during B cell maturation [45,46,47]. Homozygous deletion of mTORC1 component RAPTOR prior to lineage specification results in a total lack of B cell maturation [45,46].

Thus, mTORC1 senses both intra- and extra- cellular cues, but also the immune microenvironment, in order to influence the differentiation and maturation of immune cells.

## 4. HIV-1 Modulates mTORC1 Activity

HIV-1 infection, similarly to other viral infections, generally increases mTORC1 activity both in productively-infected and bystander host cells, thus promoting successful viral integration and replication [48,49,50,51] (graphical abstract). Moreover, mTORC1 activity is required for optimal synthesis of viral proteins, e.g., Gag [52]. mTORC1 is also activated following HIV-1 infection in peripheral blood mononuclear cells and in several model cell lines such as Jurkat cells, HeLa and HEK293 [48,49,52,53]. Nevertheless, HIV-1 is not able to sustain mTORC1 activity in conditions of nutrient deprivation or upon pharmacological inhibition of mTORC1.

When amino acids are present, GTP-loaded RagA and RagB induce mTORC1 translocation from the cytoplasm to late endosomes/lysosomes (LELs) [7]. Interestingly, Gag can also be found at late endosomes during HIV-1 egress [54]. Nutrient starvation or oxidative stress result in the accumulation of a pool of mTOR-associated LELs at a juxtanuclear position. HIV-1 is capable of redistributing perinuclear clusters of mTORC1-associated LELs [52]. This HIV-1 activity depends on the RagA and RagB, which can interact with Vif and Gag, although nothing is known about the molecular mechanisms of these interactions. Depletion of RagA and RagB reduces virus production and results in the accumulation of viral particles at the plasma membrane [52].

Although HIV-1 does not enter or replicate in neurons, its infection of brain glia cells can induce neurotoxicity and inflammation. mTORC1 activation is involved in the interaction between the proinflammatory extracellular matrix protein osteopontin in the brain and HIV-1 Env proteins, which stimulates neurite growth [55].

**Figure 2:**
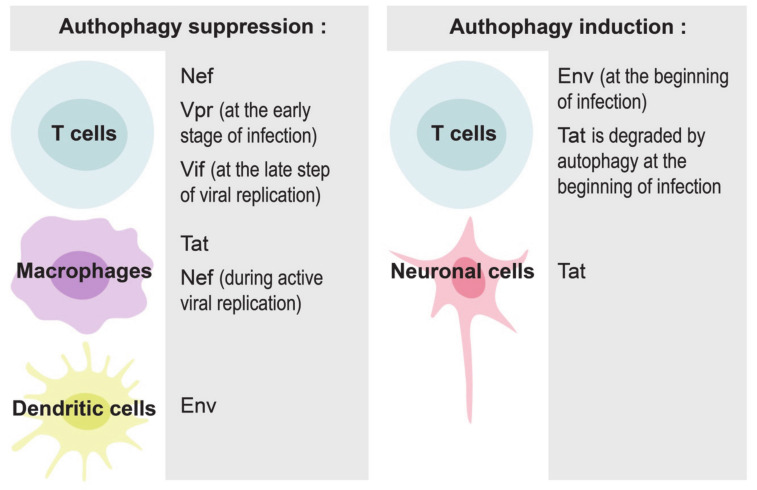
Implication of various HIV-1 proteins in the modulation of autophagy in different cells.

mTORC1 activity can be modulated both by the entire HIV-1 and by its different proteins (Figure 2). For example, mTORC1 can be activated by treatment with HIV-1 Nef, Env or Tat proteins, resulting in efficient viral replication and the generation of new virions [48,49,51,56]. In contrast, combined treatment of neuronal cells with HIV-1 Tat and methamphetamine (a stimulant drug which increases exposure to HIV-1) results in the inhibition of mTORC1 activity [57]. As Tat is present in high concentrations in patient blood serum [58,59], and because of Tat’s ability to enter almost any cell in the human body due to its cell penetration domain [60], this viral transactivator can probably remotely regulate mTORC1 activity in many noninfected cells.

## 5. Regulation of Autophagy by HIV-1

mTORC1 is the negative regulator of the major cellular catabolic process, i.e., autophagy [61,62]. Autophagy plays a critical role in maintaining cellular homeostasis, but is also related to many pathological conditions including cancer and neurodegeneration [63]. Recent studies revealed an important role of autophagy in the context of HIV-1 infection in T cells, macrophages, dendritic cells and neuronal cells [64,65] (graphical abstract, Figure 2).

Autophagy can play both pro- or anti- viral roles, depending on the stage of HIV-1 infection and the cells in contact with virus and its components. HIV-1 requires early, nondegradative autophagic events for its replication, probably because the autophagosomal membrane provides a scaffold for virus assembly [66]. The entire virus and its proteins, in particular Tat, Nef and Env, can induce autophagy in infected cells to maximize HIV-1 production. At the same time, the virus has developed multiple strategies to escape the degradation of newly-synthetized viral proteins [64]. For example, Nef can interact with BECLIN-1, an autophagy initiation protein, to inhibit autophagosome maturation in macrophages and T cells [66,67,68,69]. Nef/BECLIN-1 interaction inhibits autophagy at the transcriptional level by preventing nuclear translocation of the pro-autophagic factor TFEB in an mTORC1-dependent manner [67]. Nef can also block autophagosome formation by increasing the interaction between BECLIN-1 and its inhibitor BCL2, an interaction that requires E3-ubiquitin ligase PRKN [68]. HIV-1 proteins can also disrupt autophagy in uninfected cells. For example, HIV-1 Tat inhibits autophagy induction in noninfected primary macrophages through AKT activation [50].

At the initial steps of infection, HIV-1 envelope proteins at the surface of the virus bind to CD4 receptors, mainly CCR5, initiating autophagy in CD4 T cells [70]. This autophagic process represents an anti-HIV response of the host cell, because it selectively degrades HIV-1 Tat [71,72]. Similarly, the induction of autophagy in macrophages with dual inhibitors of PI3K and mTORC1 results in the degradation of intracellular viral particles and the reduction of viral release [73]. The virus has, however, evolved to counteract autophagy [72]. Different HIV-1 proteins participate in this inhibition in different cells and at various steps of infection. For example, Vpr tries to inhibit autophagy in T cells already at the early stages of infection to facilitate efficient viral replication [74]; Vif inhibits autophagy at the late steps of viral replication in CD4+ T cells [75]; Nef blocks autophagy in macrophages and in T cells [67,68]; HIV-1 Tat blocks autophagy in bystander macrophages [50], while envelope proteins inhibit autophagy through mTORC1 activation in dendritic cells, but not in macrophages and CD4+ T cells [56].

The regulation of autophagy was also studied in the context of HIV-1 infection in neuronal cells, as HIV-1-associated neurocognitive disorders lead to increased mortality in HIV-1 patients [76]. HIV-1 Tat induces autophagy and increases neurotoxicity [77,78,79]. HIV-1 Tat can even trigger neuronal cell death when combined with methamphetamine [57,80,81]. HIV-1 Tat also plays a role in the regulation of mitophagy, a specialized form of autophagy, which removes damaged mitochondria. Treatment with HIV-1 Tat of primary microglial cells, the key target for HIV-1 infection in the central nervous system, results in the accumulation of damaged mitochondria and increased expression of mitophagy-signaling proteins PINK1 and PARKIN. However, even though mitophagy was induced, mitophagosomes fail to fuse with lysosomes and accumulate in the cells, resulting in overall defective mitophagy and subsequent neuroinflamation [82].

## 6. mTORC1 in HIV-1 Latency 

Combination antiretroviral therapy (cART), which efficiently controls viral replication, remarkably improves the quality of life of HIV-1-positive individuals, although this therapy is not available to all people living with HIV-1, particularly in countries with limited resources. In addition, elevated risks of chronic inflammation [83], asynchronous muscle aging [84], the increased occurrence of various cancers [85,86] and developing HIV-1 drug resistance [87,88] create an urgent need to find a cure rather than to control of HIV-1 replication.

One of the major problems preventing the complete eradication of HIV-1 is the existence of latent reservoirs, “a cell type or anatomical site in which replication-competent form of virus accumulates and persists in spite of long periods of ART-suppression viremia” [89,90]. HIV-1 cellular reservoirs consist of resting memory CD4+ T cells, myeloid cells, macrophages and dendritic cells (DC) which allow the virus to survive and replicate [91]. Although the existence of anatomical HIV-1 reservoirs remains debated, the lymphoid tissues (spleen, thymus, lymph nodes and gut-associated lymphoid tissues), which are the most important sites of viral replication during infection, are considered the most prominent reservoirs.

Two opposite strategies are proposed to disrupt HIV-1 latency: “shock and kill” and “block and lock” (deep latency) [92,93]. In the “shock and kill” approach, the latent HIV-1 is reactivated (“shock”) by latency reversal agents (LRAs). The re-activated cells are further “killed” either by the attack of the host immune system or by the cytotoxic effect of HIV-1 itself, while uninfected cells are protected against viral infection by cART. Unfortunately, none of the LRAs tested so far have shown a significant effect on the viral reservoir in clinical trials [94]. In addition, agents inducing global T cell activation induced severe adverse reactions, mainly through inflammation, and were abandoned after several clinical trials. “Shocking” alone seems to be insufficient to eliminate latent T-cell reservoir. Effective “killing” strategies need to be developed to optimize this approach. An alternative “block and lock” strategy aims to bring the proviral HIV-1 to a deeply silenced state so that HIV-1 will not rebound, even if cART is discontinued. Recent findings demonstrate that manipulation of the mTORC1 pathway is important for the efficient development of both strategies.

The first notion of the importance of mTORC1 in HIV-1 latency came from the study by Besnard et al., who demonstrated that both mTORC1 and mTORC2 complexes are essential for HIV-1 reactivation from latency [95]. Accordingly, inhibitors which block both mTORC1 and mTORC2, pp242 and Torin1, prevent HIV-1 reactivation in primary CD4+ T cells from uninfected donors when infected ex vivo [49,95]. Rapamycin, which mostly blocks mTORC1, is a less effective inhibitor of HIV-1 reactivation. Moreover, in CD4+ T cells from aviremic HIV-1-positive donors under cART, HIV-1 proviral reactivation is unaffected by the addition of rapamycin, even if the drug downregulates markers of toxicity related to inflammation [96]. At the same time, active mTORC1 might be important for natural HIV-1 suppression control, because mTORC1 activation was also detected in HIV-1 elite controllers, a rare (<1%) group of HIV-1-infected patients who do not take antiretroviral therapy (ART) and do not develop AIDS [97].

The use of rapamycin and pan-inhibitors of mTORC1 is associated with multiple adverse effects; therefore, targeting the mTORC1 signaling pathway and not mTOR kinase itself appears to be an attractive alternative approach. Recent genome-wide CRISPR screening for the host factors required for HIV-1 latency identified two upstream mTORC1 inhibitory genes: TSC1 and DEPDC5; both are known negative regulators of mTORC1, but they suppress mTORC1 via different upstream branches. TSC1, a TSC complex member, inhibits mTORC1 and maintains HIV-1 latency by downregulation of RHEB, whereas DEPDC5, GATOR1 component, does so via suppression of RagA [98]. Therefore, targeting of TSC1 and DEPDC5 might be a useful strategy in metabolic inhibition to “block and lock” the latent reservoir.

In an effort to optimize the “shock and kill” approach, recent findings revealed that interaction of infected CD4+ T cells with DC could activate latent HIV-1 [99]. The contact with dendritic cells also activates the PI3K-Akt-mTOR pathway in CD4+ T cells and contributes to HIV-1 purge.

CD4+ T cells express on their surface various receptors that target them to different peripheral tissues in the body. One such receptor, CCR6, directs Th17 T-helper cells to the gut. The gut-associated lymphoid tissues are considered an important site of HIV-1 replication and a viral reservoir. The analysis of colon biopsies taken from HIV-1 patients under cART revealed that CCR6+ Th17-polarized CD4+ T cells express more mTOR, and that mTOR phosphorylation is also increased in these cells [53]. The use of mTOR inhibitors limited HIV-1 replication in gut-homing Th17 cells during reverse transcription and prior integration, with mTORC1/mTORC2 inhibitor INK128 being more effective than rapamycin. Thus, mTOR inhibitors could have a potential beneficial effect in decreasing HIV-1 reservoirs and restoring Th17 immunity in intestines during cART.

Finally, recent mathematical models predict that the persistence of a majority of HIV-1 infected cells is due to cellular proliferation rather than HIV-1 replication [100]; therefore, reducing cell proliferation could decrease the size of the HIV-1 reservoir. mTORC1 is a major regulator of cellular proliferation, and the specific targeting of this pathway in HIV-1 latent cells appears to be a promising approach by which to cure AIDS.

## 7. mTORC1 Pathway in HIV-1-Related Diseases

**Figure 3:**
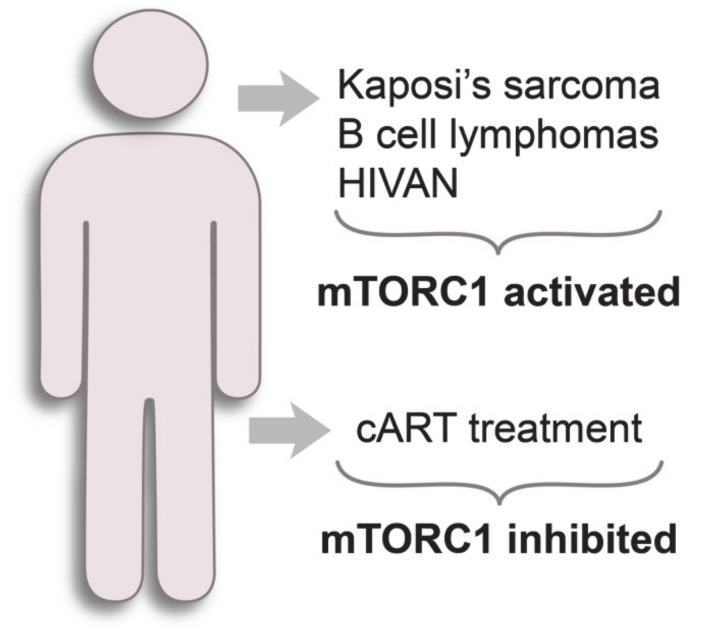
mTORC1 status in HIV-1-related diseases.

HIV-1-infected individuals have an elevated risk of developing of so-called AIDS-defining cancers: Kaposi’s sarcoma, B-cell lymphomas and cervical cancer [101] (Figure 3). Current antiretroviral therapy efficiently suppresses HIV-1, but high incidence of HIV-1-associated malignancies persists, although this has sharply declined in developed countries since the introduction of cART [86]. Thus, it is important to understand the mechanisms of the development of HIV-1-related diseases, even in patients under cART.

Kaposi’s sarcoma is caused by a herpesvirus (KSHV or HHV8). Ectopic expression of the viral proteins K1 and vGPCR in primary human umbilical vein endothelial cells (HUVEC) was shown to activate PI3K-AKT-mTOR pathway, which plays a central role in Kaposi’s sarcomagenesis [51,102,103,104]. KSHV infection selectively upregulates mTORC1 signaling in primary human endothelial cells (LECs), but not in blood endothelial cells [105]. KSHV infection of LECs makes these cells dependent on the mTORC1 pathway for their survival, because the treatment of these cells with rapamycin triggers apoptosis. Interestingly, primary effusion lymphoma, a tumor of B cell origin linked to KSHV infection, was strongly inhibited by rapamycin both in vitro and in mouse models [106].

Although HIV-1 does not infect B lymphocytes, the most frequent AIDS-related lymphomas (ARLs) are always of B cell origin [107]. One of the reasons for these malignancies might the genomic instability and chromatin remodeling in B cells caused by the entire HIV-1 and HIV-1 Tat protein, which is present in the blood of HIV-1-infected individuals and can penetrate different cells, including B cells [59,108]. Another possibility might involve the mTORC1 pathway, because studies in cell lines, primary cultures and tissue samples from patients have shown that mTORC1 is hyperactivated in diffuse large B cell lymphoma (DLBCL), mantle cell lymphoma (MCL), Hodgkin lymphoma (HL), Burkitt lymphoma (BL) and anaplastic large cell lymphoma [109,110,111,112,113,114]. In order to inhibit mTORC1, rapalogues were used in clinical trials in MCL, DLBCL, HL and indolent lymphoma, but failed to provide substantial benefits for patients [115,116,117,118,119]. This failure is not specific to B-cell malignancies, but also concerns many other cancers. The main reason for rapalogue inefficiency is related to the activation of alternative proliferation pathways and genetic and functional intratumoral heterogeneity of mTORC1 activity [120]. Recent advances in the development of alternative mTOR inhibitors (pan-inhibitors and dual inhibitors) and the results of combination therapies with DNA damaging anticancer drugs should bring an essential breakthrough in the treatment of these cancers [8]. Indeed, the simultaneous inhibition of Akt with Nelfinavir and MK-2206 and mTORC1 with rapamycin showed a synergistic effect in suppressing DLBCL [113,121].

Similarly, the combination of mTORC1 inhibitors with different anti-HIV-1 drugs showed significant effects in suppressing viral replication, even rendering the antiviral drug-resistant strains sensitive to medication in T cells, PBMCs and in a humanized mouse model [15,48,122,123].

One of the frequent complications occurring typically in young adults of African ancestry with advanced HIV-1 is a HIV-associated nephropathy (HIVAN). The role of mTORC1 in HIVAN was addressed both in HIVAN murine models and upon analysis of tissue samples obtained from renal cortical sections of HIVAN patients [124,125,126,127,128]. Both HIVAN mice and HIVAN patients exhibit the activation of the mTORC1 pathway, as shown by elevated phosphorylation of mTOR itself and its substrates p70SK1 and 4EBP1. Treatment with rapamycin not only attenuated the HIVAN phenotype, but also partially inhibited tubular cell protein synthesis, specific miRNA expression and renal lesions [124,125,126]. In addition, inhibition of the mTORC1 pathway in HIVAN mice resulted in the downregulation of renal tissue p53 expression, and provided protection against p53-mediated oxidative kidney cell injury [127].

Besides the suppression of HIV-1 replication, cART drugs have several additional pharmacological activities that interfere with the mTORC1 pathway. A canonical cART is a combination of two nucleoside reverse transcriptase inhibitors (NRTIs) with one of the following drugs: HIV-protease inhibitors (PIs), nonnucleoside reverse transcriptase inhibitors (NNRTIs) or HIV-integrase strand transfer inhibitors (INSTIs) [129]. Other FDA-approved classes of antiretroviral drugs are fusion inhibitors, CCR5 antagonists, CD4 post-attachment inhibitors [129]. PIs act via different mechanisms, mainly associated with the inhibition of cellular proteasome machinery (Hsp90 inhibition, ER stress). This leads to decreased PI3K-AKT signaling and downstream mTORC1 downregulation [130,131,132] (Figure 3). CCR5 antagonists also disrupt the PI3K-AKT-mTORC1 pathway [133,134]. In contrast, the side action of NRTIs can implicate active mTORC1 signaling in the host cell, although a direct link between them has not yet been established. The administration of NRTIs may cause severe mitochondrial toxicity [135], related to their moderate affinity to host DNA polymerases, especially the mitochondrial polymerase γ [136]. Mitochondrial toxicity can lead to the inhibition of late stages of autophagy mediated by active mTORC1 signaling, as shown for zidovudine (NRTI) in myocytes [137]. Another study demonstrated that stavudine (NRTI) could activate mTORC1 signaling in a mouse model of peripheral neuropathic pain [138]. Treatment with rapamycin attenuates persistent neuropathic pain in mice. Additionally, several cART drugs, being substrates of the CYP450 enzyme family, can modify the metabolism of mTORC1 inhibitors when used concomitantly, leading to altered mTORC1 axis regulation in patients [139].

## 8. Concluding Remarks

As a central integrator of various stimuli from intra- and extra- cellular environments, mTORC1 plays a vital role both in physiological and pathological conditions, including viral infections and cancers. In the immune system, mTORC1 senses cues from the immune microenvironment and influences the differentiation and maturation of immune cells. During HIV-1 infection, the mTORC1 pathway can be modulated not only by the entire virus, but also by its proteins. A combination of anti-HIV drugs with inhibitors of the mTORC1 pathway might be an efficient measure to cure, rather than control, HIV-1 replication.

## 9. Outstanding Questions

What are the molecular mechanisms of the interaction of HIV-1 and its separate proteins with the components of the mTORC1 pathway?

Are there ways to efficiently and specifically control mTORC1 suppression (and autophagy activation) in host cells?

Will a new generation of mTORC1 inhibitors provide additional benefits in overcoming of HIV-1 latency?

To what extent is the mTORC1 pathway involved in the appearance and persistence of HIV-related malignances in patients under cART?
